# Profound and pervasive degradation of Madagascar’s freshwater wetlands and links with biodiversity

**DOI:** 10.1371/journal.pone.0182673

**Published:** 2017-08-08

**Authors:** Andrew J. Bamford, Felix Razafindrajao, Richard P. Young, Geoff M. Hilton

**Affiliations:** 1 Wildfowl & Wetlands Trust, Slimbridge, Gloucestershire, United Kingdom; 2 Durrell Wildlife Conservation Trust, BP, Antananarivo 101, Madagascar; 3 Durrell Wildlife Conservation Trust, Les Augrès Manor, Trinity, Jersey, United Kingdom; 4 Department of Life Sciences, Imperial College London, Silwood Park Campus, Ascot, Berkshire, United Kingdom; University of Sydney, AUSTRALIA

## Abstract

Reflecting a global trend, freshwater wetlands in Madagascar have received little conservation or research attention. Madagascar is a global conservation priority due to its high level of species endemism but most work has focused on protecting forests. For the first time, we investigated the state of wetlands across the country to determine the effects of human disturbance. We conducted a rapid survey of 37 wetlands, using waterbirds and benthic invertebrates as ecological indicators. We recorded nine variables relating to human disturbance, revealing widespread wetland destruction. Principal Components Analysis reduced the nine variables to a single Principal Component (PC) that explained 50% of the dataset variance, demonstrating that different forms of human disturbance are ubiquitous and inseparable. The disturbance PC provides an index of how pristine a lake is and in Generalized Linear Models (GLMs) was significantly inversely related to the number of waterbird species present and the density of Chironomidae. The disturbance PC was estimated for every wetland in a GIS-derived dataset of wetland locations in Madagascar, giving a country-wide frequency distribution of disturbance. To validate the estimated PC values, we used the GLMs to predict the number of endemic bird species at an independent sample of 22 lakes. The predicted values correlated with the observed number of species, demonstrating that our procedure can identify lakes with high biodiversity value. The disturbance PC provides a convenient method for ranking sites, and a country-wide ranking demonstrates that the only near-pristine lakes in Madagascar are small sites that have been preserved by remoteness from human activity and not conservation management. The strategy of conserving high biodiversity remnants is insufficient because existing remnants suffer some degree of degradation and only support small populations of threatened species. Large-scale restoration of degraded wetlands is required for the long-term conservation of Madagascar’s freshwater biodiversity.

## Introduction

Freshwater ecosystems are the most threatened major habitat type globally [1], with freshwater vertebrate species declining faster than those in either terrestrial or marine realms [2]. Despite this, conservation research and investment in freshwater habitats is disproportionately low [3], with tropical wetlands particularly threatened and under-researched [4].

The main threats to wetlands globally are overexploitation of wetland resources including fish, pollution from agricultural and industrial processes, flow modification to provide water for cities and especially crops, habitat destruction or degradation, invasive species and climate change [5–7]. All of these problems are exacerbated by rising human populations, and tropical areas are seeing the highest rates of population growth and agricultural growth [7]. Tropical countries are also among the least well placed to deal with these issues due to generally very low institutional capacity [8].

Madagascar provides a good example. The country is of immense conservation interest for its high level of species endemism and threat, but activity has focused almost entirely on forests. Of the 46 Strict Reserves, National Parks and Special Reserves established before 2011, 45 protect forest ecosystems. As a result, Madagascar is losing wetlands faster than forest. Since 1960, the highland regions have lost 60% of wetlands, compared to 20% of forests [9].

Madagascar’s wetlands contain fewer iconic species than the forests, but show similar rates of species endemism [10,11] and are at least equally threatened. Half of the native freshwater fish species and half of the freshwater amphibians are classified as vulnerable or worse in the IUCN Red Data list [12]. Of Madagascar’s 12 bird species classified as Endangered or Critically Endangered, 9 are wetland birds. The only recent documented extinctions have been of freshwater species, the Alaotra grebe (*Tachybaptus rufolavatus*) and three fish species.

Of the major threats to wetlands globally [5], not all are significant problems in Madgascar. Flow modification is a minor problem and the level of pollution is unknown in general, although it is high at some sites. Habitat degradation is a major concern, with two forms predominating–marsh clearance for rice farming and siltation caused by high rates of soil erosion from deforested land. Conversion to rice farming is the main cause of the loss of natural wetlands [9]. Invasive species include fish (tilapia, *Oreochromis*, *Sarotherodon* and *Tilapia* species, common carp, *Cyprinus carpio*, and Asian snakehead, *Channa* cf. *striata*) and plants (water hyacinth, *Eichornia crassipes*, and *Salvinia molesta*), although little is known about the effect they may have on aquatic ecosystems [13].

Madagascar is one of the poorest countries in the world and livelihoods are often wetland dependent. Half of the population, and 65% of the rural population, are dependent on unimproved water sources [14], such as rivers and lakes. Wetlands also supply most of the country’s staple foodstuff, rice, the majority of which is grown in wetlands cleared of their natural vegetation, either in lowland rain-fed systems or irrigated systems planted on alluvial soils [15].

Given the imbalance in protection between forests and wetlands, we aimed to investigate for the first time the status of wetlands across the country and use this information to assess whether the existing extremely low level of conservation protection is sufficient to protect remaining freshwater biodiversity. There is little monitoring of wetlands in Madagascar and little information on the condition of the remaining habitat. Our objectives are 1) to quantify the extent of wetland degradation across country and; 2) to look for links between human disturbance of wetlands and biodiversity. We conducted a rapid survey of wetlands to assess biodiversity, using birds and benthic invertebrates as indicators, and related these results to disturbance variables measured in the field and using remote sensing data. We then extrapolated these results to predict the condition of all lakes within Madagascar.

## Methods

### Site selection

We created a GIS dataset of lakes in Madagascar based on a water cover dataset [16]. To improve the spatial resolution of the dataset, the Spatial Analyst extension in ArcGIS v10 was used to identify possible lakes by predicting sink areas and areas with zero slope, based on Digital Elevation Model data [17]. The resultant coverage was compared with LandSat images (available from the U.S. Geological Survey https://www.usgs.gov/) and manually cleaned. The result is that all lakes in the dataset (a total of 973) are confirmed from satellite images, but not all lakes in the country will be in the dataset. In particular, due to the spatial resolution of the input data, very small lakes (under 2 ha.) could be missed.

**Fig 1:**
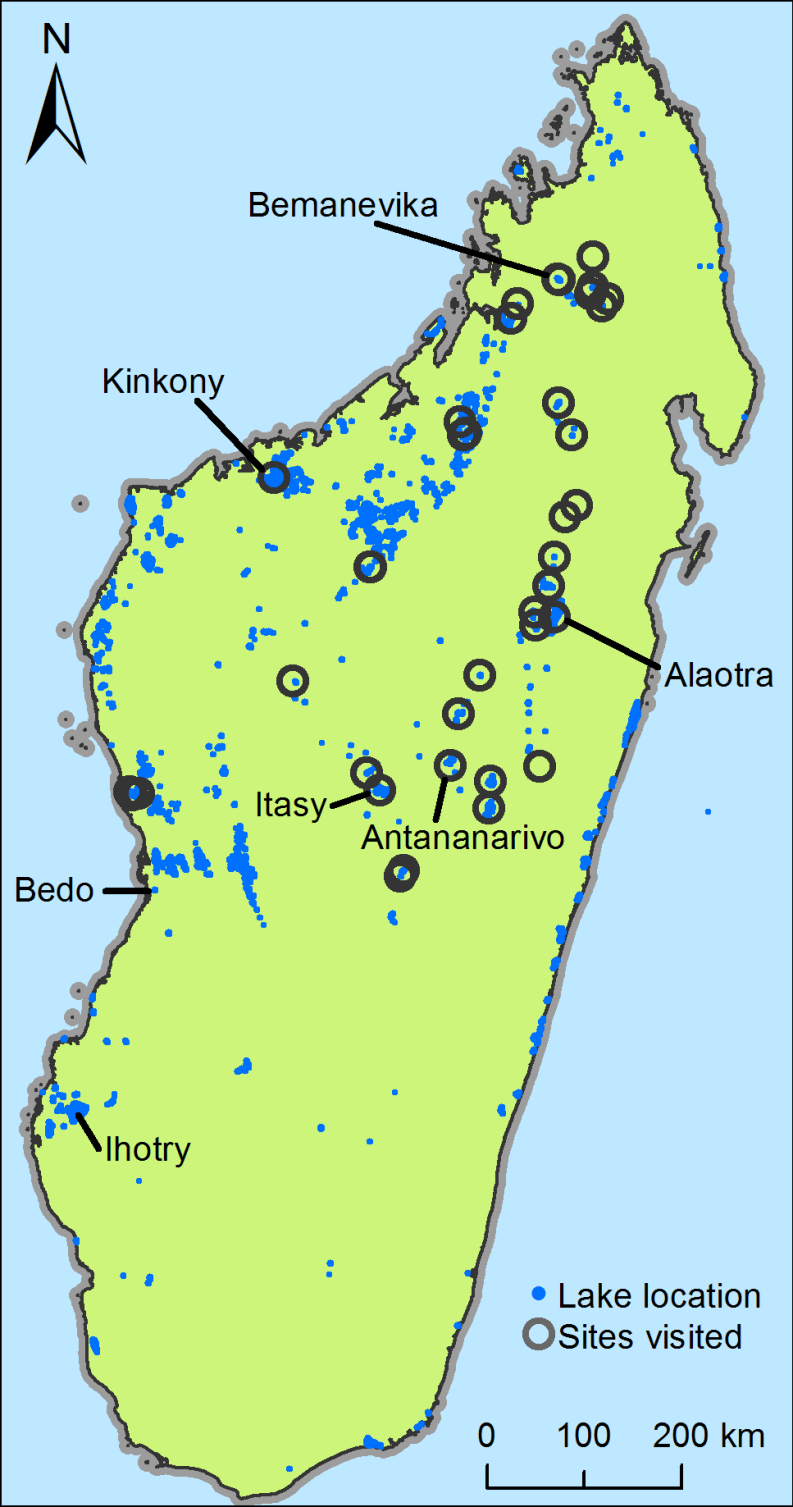
Map of wetlands in Madagascar. The locations of 973 lakes predicted in our GIS dataset and the 37 wetland sites that were visited in this study. Other sites referred to in the text are highlighted.

This dataset of lakes was used to inform the choice of survey sites. We focused on freshwater lakes in the central and northern highlands and west coast regions. Our survey did not cover the acidic and brackish east coast wetlands, nor did we visit any sites in the southern highlands or south-west coastal region where the few lakes present tend to be brackish and are relatively well studied. Selection of lakes for surveying was biased towards the larger lakes shown in our GIS dataset and lakes that we had reason to suspect (based on past surveys, existing conservation projects or local advice) might contain high levels of biodiversity (Fig 1).

### Biodiversity data

We visited a total of 37 lakes in 25 catchments, including nine lakes in four Ramsar sites. All visits took place between October 2011 and December 2013, with the exception of one site visited in June 2015. Nine lakes were subject to repeat visits, each being visited on three separate occasions (see Statistical Analyses section). Visits lasted between four and eight hours, longer at larger lakes, starting between 06:00 and 08:30. All visits were made by the same two people, AB and FR. We recorded information on the presence of waterbirds and benthic invertebrates, both commonly used indicators of the health of wetland ecosystems [18]. Birds were recorded throughout the visit, and we also asked fishermen about the species present on the lake. Due to the limited effort that could be put into the survey, we focused on birds associated with open water as they are easier to detect than marsh birds. The analysis therefore focusses on birds in the families Podicipedidae, Ardeidae, Anatidae and Jacanidae and also includes three species of Railidae, Common Moorhen (*Gallinula chloropus*), Red-knobbed Coot (*Fulica cristata*) and Sakalava Rail (*Zapornia olivieri*), and one species of Accipitridae, Madagascar Fish Eagle (*Haliaeetus vociferoides*), as it is both wetland dependent and very easy to detect.

Benthic sediment samples were taken from a boat using a petit-ponar grab sampler (WildCo., Florida) with a sampling area of 0.0231 m^2^. We collected at least five samples per lake and more samples (max. of 15) from larger lakes. Sample locations were mostly random, although we did sample close to any marginal or emergent vegetation if present, and spread out over the entire lake. Substrate type was noted, categorized as either sand, silt or clay. We sieved samples to remove sediment, spread them in a tray and searched for 5 minutes. This search time was selected after trialing longer search times. Extra search effort beyond 5 minutes did not result in larger invertebrate counts regardless of substrate type, and consequently we are reasonably certain that we could identify virtually all invertebrates present in a sample within this search time. Specimens visible to the naked eye were collected and identified to taxonomic order, with the exception of molluscs which were identified only to class (i.e., gastropod or bivalve). Counts of specimens in the orders Ephemeroptera, Plecoptera and Trichoptera (EPT) were combined as together they are a common indicator group for freshwater systems, and Chironomidae were counted separately from other Diptera as they are a common indicator group for lakes [18].

### Human disturbance data

Nine variables representing human disturbance were used in the analysis (Table 1). One variable (how many, out of five, common invasive species were present) was recorded during site visits. Data for five variables (sedimentation, population density in the watershed, population density at the lake, forest cover in the watershed, forest cover surrounding the lake) were extracted from pre-existing GIS datasets and the remaining three (rice cover in the watershed, ratio of rice to natural marsh vegetation, ratio of rice to lake size) were derived from satellite images and ground-truthed during site visits. All the variables were structured so that an increase in value represents a more disturbed lake.

**Table 1:** Human disturbance variables used in the analysis.

Variable	Temporal coverage	Description
*Water quality*		
Sedimentation	DEM from 2000 Vegetation data 2003–2006	Tons km^-2^. Estimated using the Revised Universal Soil Loss Equation (RUSLE), following Maina et al. [19], based on DEM and vegetation atlas data [17,20].
*Agriculture*		
Rice agriculture relative to marsh area	Landsat images from 2000, corrected during site visits	A measure of marsh clearance, based on the assumption that rice farming methods used in Madagascar rely on clearing natural wetlands. Areas of rice and marsh were measured from LandSat images* and ground-truthing during site visits. The area of rice was divided by the total area of rice and marsh.
Rice agriculture relative to lake area	Landsat images from 2000, corrected during site visits	Measured from LandSat* images and ground-truthing during site visits. The area of rice was divided by the surface area of the lake.
Rice cultivation in watershed	Landsat images from 2000, corrected using Google Earth images (image dates 2011–2015, accessed May 2016)	The proportion of the watershed covered by rice agriculture. For small watersheds this was directly measured from LandSat* images; for larger areas, images were sampled at 100 random point locations to give an estimate.
*Vegetation*		
Invasive species	Recorded during site visits	A score out of 5 based on the presence or absence of five common invasive organisms. Two plants (*Salvinia molesta* and *Eichhornia crassipes*) and three fish (*Cyprinus carpio*; *Channa* cf. *striata*; and tilapia, *Oreochromis*, *Sarotherodon* and *Tilapia* species). To determine the presence of non-native fish, local fishermen were interviewed in Malagasy. The score was treated as a continuous variable, which is not ideal but tolerable as the presence of each species is regarded as equal.
Non-forested land in watershed	DEM from 2000 Vegetation data 2003–2006	The proportion of the watershed that is not covered by forest. Watersheds were calculated using the Spatial Analyst extension to ArcGIS based on DEM data [17]. Forest cover taken from a vegetation atlas [20].
Non-forested land surrounding the lake	DEM from 2000 Vegetation data 2003–2006	The proportion of a 200m buffer surrounding the lake that is not covered by forest.
*Population*		
Human population density in watershed	Madagascar data from 2010	The mean human population density (people km^-2^) over the entire watershed [21].
Human population density at the lake.	Madagascar data from 2010	The mean human population density (people km^-2^) in a 200m buffer surrounding the lake [21].

* LandSat images are available from the U.S. Geological Survey https://www.usgs.gov/.

### Statistical analyses

All analyses were carried out using R 3.2.4 [22], using the package MASS [23]. We used Principal Components Analysis (PCA) on the human disturbance variables using the command princomp(). Variables were log-transformed as they were strongly skewed and the PCA was carried out using the correlation matrix, suitable in cases where the variables have very different scales.

We used the first four resulting Principal Components (PCs) as explanatory variables in Generalized Linear Models (GLMs) of the occurrence of taxa recorded in the surveys. We modeled the number of invertebrate taxa (i.e. the total number of orders present from all samples in a lake), the total number of waterbird species and the number of endemic waterbird species recorded using log-linear regression. For specific invertebrate taxa, and the most commonly recorded endemic bird species–Meller’s duck (*Anas melleri*) and Madagascar grebe (*Tachybaptus pelzelnii*)–we used recorded presence or absence at each lake as the response variable in logistic regressions. The only exception to this was Chironomidae, which were recorded in nearly all visited lakes, making presence-absence models unsuitable. For Chironomidae we modelled the mean number of individuals per sample using a negative binomial model. To control for sampling effort, the number of samples taken was included in all invertebrate models and the total survey time was included in all bird models.

Due to the difficulty in accessing some of the sites and logistical constraints, repeat surveys were not possible at many of the sites. However, false negatives–failing to record a species that is present–can have significant effects on habitat analyses [24]. The danger here is that if detectability of species varies with human disturbance, this will affect estimates in the GLMs of the influence of disturbance. We attempted to verify our results by re-analyzing the subset of nine sites that were visited multiple times, along with six sites that had been surveyed before (by FR in 2007), giving us 15 sites that were visited on three occasions (S1 Table). To estimate occupancy and detection rates for *A*. *melleri* and *T*. *pelzelnii*, we used a single-season model [25] implemented in the program PRESENCE [26]. We compared four models: 1) Assuming constant occupancy and detection rates across sites; 2) Constant occupancy, detection varying with PC1; 3) Occupancy varying with PC1, constant detection; 4) Both occupancy and detection varying with PC1. We used Akaike’s Information Criterion (AIC) to select the best model in each case.

### National wetland analysis

To predict the condition of lakes that we did not visit, we created a reduced set of habitat variables comprising the five variables derived entirely from pre-existing GIS datasets (excluding the three rice measurements and the count of invasive species), and carried out a second PCA for the visited sites using this reduced set of variables. The outputs of the two PCAs were compared to see if the subset of variables could be used to estimate the full set. We then calculated Principal Components for all lakes predicted in our GIS model. To verify these predictions, we used data from several published bird surveys conducted in the past 15 years [27–29] and data from one additional site that we surveyed in Nov 2016 (S4 Table). From each survey we extracted the number of endemic bird species associated with open water that were present, giving us species counts at 22 lakes in the west coast and south-west regions. We then used the parameter estimates from the bird species GLM to predict the number of species present based on our predicted Principal Components. The predictions were compared to the observed number of bird species using Spearman’s rank correlation test.

## Results

### Wetland surveys

In total, 257 benthic sediment samples were taken from 37 lakes. The majority of samples taken in the central and northern highlands were silt (129 out of 181 samples), whereas clay was the dominant substrate in the western coastal region (45 out of 76). Only 22 samples were sand. Twelve invertebrate taxa were recorded overall, a mean of 2.8 taxa per lake. The most common taxon was Diptera (the majority of which were Chironomidae), followed by Gastropoda. Only seven lakes contained any EPT and only four lakes contained bivalves. The results for each lake visited are in S1 Table.

The most common birds recorded were red-billed teal *Anas erythrorhyncha* and white-faced whistling duck *Dendrocygna viduata* recorded on 19 and 25 lakes respectively. Endemic water birds were seen on only 14 lakes. The most commonly seen endemic was *A*. *melleri*, on nine lakes.

### Disturbance and biodiversity analysis

**Fig 2:**
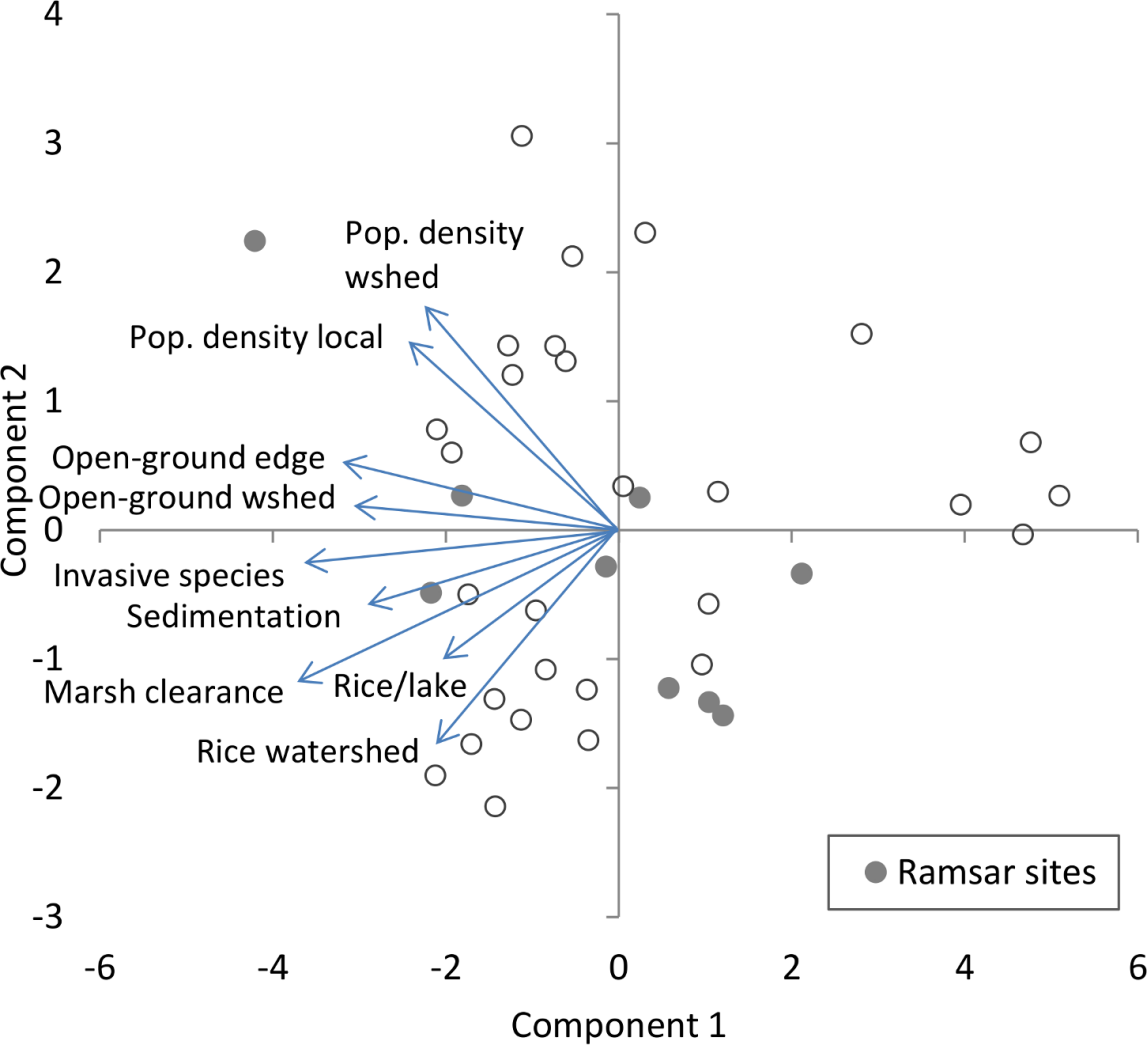
Biplot of principal components analysis of human disturbance variables at wetland sites in Madagascar. The nine variables relating to human disturbance were log-transformed and the PCA based on the correlation matrix due to their very different scales.

The human disturbance data revealed high levels of disturbance across Madagascar. In total, 82% of marsh at the sample sites had been cleared for agriculture. Overall land cover in the watersheds was 9% agriculture and 18% forest cover. The various forms of human disturbance measured were all associated with each other, as the nine variables measured were reduced to four components that explained 89% of the variance (S2 Table). PC1 alone explains 50% of the variance. In general, PC1 (Fig 2) is a measure of how pristine each lake is, being strongly negatively correlated with all nine disturbance variables: high values are associated with little rice cultivation, few people, low sedimentation, few invasive species and little marsh or forest clearance. High values of PC2 (Fig 2) are associated with lakes that are unaffected by rice agriculture but nevertheless have many people around them–generally lakes in urban areas–while low values are associated with lakes that have rice agriculture but a low population density.

**Fig 3:**
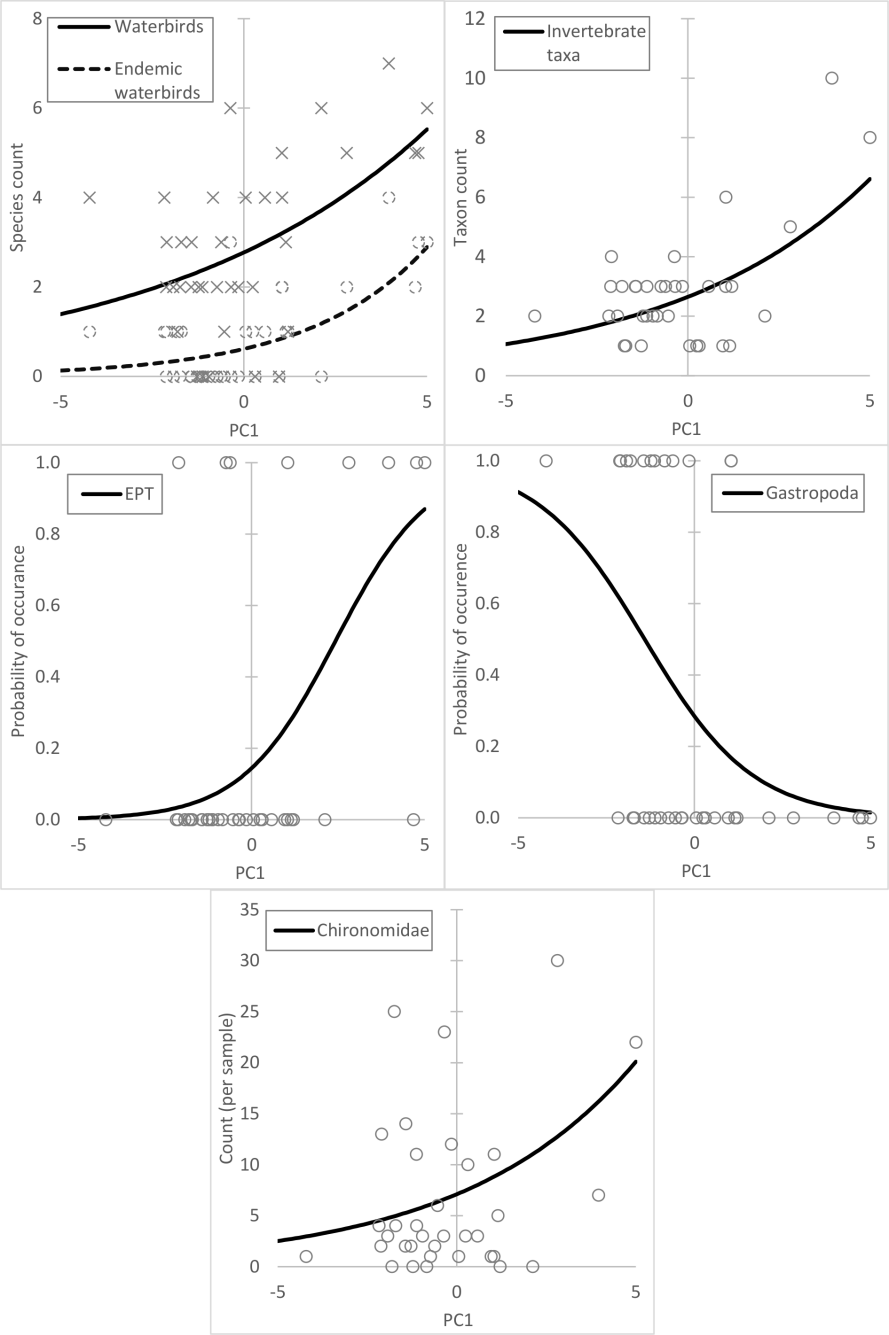
Relationship between PC1 and biodiversity recorded at lakes. Fitted values from GLMs across the range of values for PC1 recorded at the 37 surveyed lakes.

There were significant associations between PC1 and the number of invertebrate and bird taxa (Table 2, Fig 3), but other principal component axes were not significantly associated with biodiversity. The overall numbers of both invertebrate taxa and endemic bird species were higher in lakes with larger values of PC1. Abundance of Chironomidae and presence of EPT, *A*. *melleri* and *T*. *pelzelnii* were also associated with larger values of PC1. Conversely, presence of gastropods was associated with lower values of PC1.

**Table 2:** The effects of human disturbance on freshwater birds and invertebrates. Results of GLMs using the first Principal Component (PC1) from a PCA analysis of human disturbance of wetland sites to explain occurrence of benthic invertebrates and waterbirds.

Taxon	Co-efficient	F	d.f.	P
*Taxon richness (Log-linear models)*				
Invertebrates	0.19	-14.1	33,1	<0.001
Waterbirds	0.14	-11.1	35,1	<0.001
Endemic waterbirds	0.31	-18.3	35,1	<0.001
*Abundance (Negative binomial model)*				
Chironomidae	0.21	-4.2	33.1	0.04
*Presence / absence (Logistic models)*				
Chaoboridae		-1.6	34,1	0.2
EPT	0.73	-9.8	34,1	0.002
Oligochaetae		0.0	34,1	0.9
Gastropoda	−0.65	-10.5	34,1	0.006
*Anas melleri*	1.30	-18.9	35,1	<0.001
*Tachybaptus pelzelnii*	0.99	-16.7	35,1	<0.001

The patch occupancy estimates for *A*. *melleri* and *T*. *pelzelnii* at the subset of the lakes that were visited on multiple occasions were consistent with the results in Table 2. For both species, the best model suggested that both occupancy and detection rate were positively related to PC1, although in the case of *A*. *melleri* this model was only marginally better than the model in which the occupancy rate altered and detection rate was constant (Table 3). Hence, this confirms that both species were less likely to occur on more disturbed lakes, but additionally suggests that they are less likely to be seen on such lakes.

**Table 3:** AIC values for occupancy models. AIC values for patch occupancy models of two bird species at 15 lakes. Models allowed the probability of occupancy (ψ) and the probability of detection (*p*) to vary with human disturbance. The best models are in bold.

		*A*. *melleri*		*T*. *pelzelnii*	
Model	d.f.	AIC	Akaike weight	AIC	Akaike weight
Constant ψ and *p*	2	42.8	0.10	30.4	0.00
ψ alters with PC1, constant *p*	3	**40.2**	**0.37**	23.0	0.18
Constant ψ, *p* alters with PC1	3	42.0	0.15	22.2	0.28
ψ and *p* alter with PC1	4	**40.1**	**0.39**	**20.9**	**0.53**

### National wetland analysis

**Fig 4:**
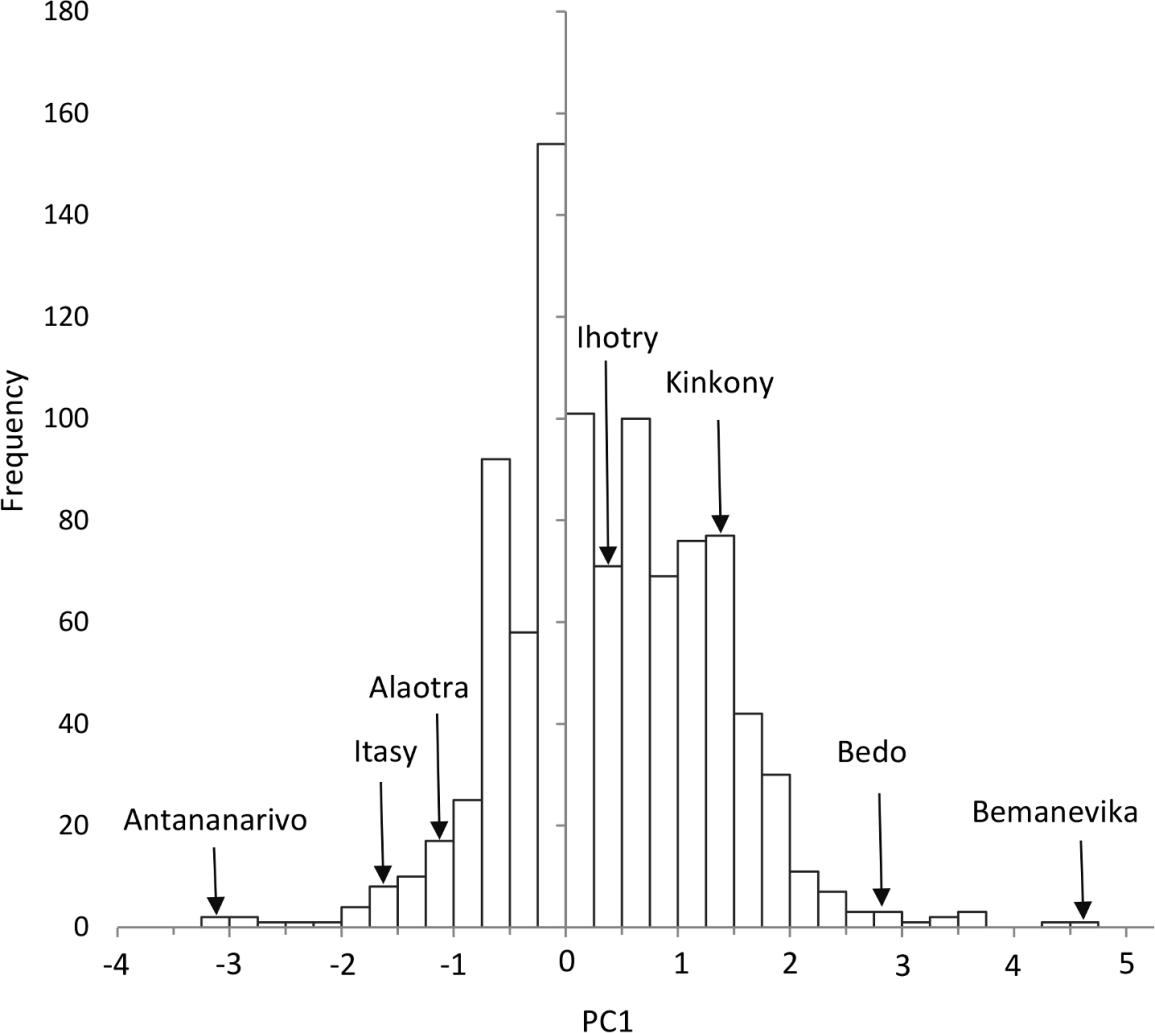
Frequency distribution of human disturbance scores for all lakes in Madagascar. Values of PC1 from a Principal Components Analysis of human disturbance data, calculated for the 973 lakes in a database of wetland locations in Madagascar. Some well-known lakes are highlighted to help calibrate the scale.

In the reduced PCA using only the five GIS derived disturbance variables, PC1 explained 62% of the variance (S2 Table). The reduced PC1 correlated strongly with PC1 from the full PCA (Pearson’s correlation, r^2^ = 0.9, p<0.001) and therefore serves as a proxy measure of disturbance for unvisited lakes in Madagascar. The reduced PC1 was calculated for all 973 lakes shown in our GIS model (S3 Table). The frequency distribution of scores is shown in Fig 4 with some well-known sites highlighted for calibration. The model predicted the Bemanevika wetlands in the northern Highlands to be the least disturbed lakes in the country; the various lakes in Antananarivo were, unsurprisingly, predicted as the most disturbed. The modelled number of bird species at the 22 test sites correlated significantly with the observed number of species (S4 Table, Spearman’s rho = 0.51, P = 0.01) demonstrating that the model can identify sites of high biodiversity value.

## Discussion

Our results demonstrate the profound scale of wetland degradation in Madagascar. We found few lakes free of human disturbance and a substantial majority that supported few endemic birds and contained extremely low abundance and diversity of benthic invertebrates. Furthermore, we found a clear link between human disturbance of wetlands and the measured aspects of biodiversity found in those wetlands.

Species counts of waterbirds were lower in more disturbed wetlands, as was abundance of Chironomidae, a group often used to indicate the condition of lakes [18]. Taxon counts of benthic invertebrates were also lower in disturbed lakes, as was the likelihood of Meller’s duck, Madagascar grebe and EPT being present. In contrast, Gastropoda were associated with more disturbed lakes. Most of the gastropod specimens that we collected appeared to be of the same species. Specimens from lakes Itasy and Kinkony were identified as *Melanoides tuberculatus* (S. Brooks, pers. comm.), a species known to be resistant to pollution [30] and a problematic invasive species in the Americas, although it is native to Madagascar. The basic survey methods that we used mean that care should be taken in interpreting these results. We uncovered a potentially confounding effect of lower detectability of some bird species at more disturbed lakes, which may have affected the results for birds. Most importantly, use of greater taxonomic resolution in the invertebrate surveys may have produced differing results, as the response to disturbances of particular species or genera of Chironomidae or EPT may have differed from the overall trends recorded here. The fact that overall trends were apparent suggests that each group was dominated by a particular species or taxa, as was the case for the Gastropoda and *Melanoides*. Surveys that identify invertebrates to species level or close may well reveal more about what human pressures are causing the very low invertebrate abundance recorded in our surveys. Undertaking a more detailed analysis of this type would, however, have prevented us from obtaining the breadth of coverage that we did. Overall, there is undoubtedly a need for more detailed research, but the trends that we have uncovered are very clear.

Other taxa were recorded too infrequently to analyse links with human disturbance, although there is some reason to think they may also be affected by disturbance. The historic decline of the Critically Endangered Madagascar pochard (*Aythya innotata*) [31] appears unsurprising in the context of our results–the one site at which it occurs was the least disturbed site in our survey. However, despite the clarity of our results, we cannot be sure that those aspects of biodiversity that we were able to measure in our brief surveys will act as good indicators for other taxa of concern, notably fish, and this is an area that urgently requires investigating.

It is difficult to disentangle the effects of different pressures on Madagascan lakes, as demonstrated by the Principal Components Analysis in which PC1 explained 50% of the variance in the dataset. It seems that the pressures on Madagascan lakes are ubiquitous and hence inseparable. PC1 provides a convenient method for ranking lakes by disturbance level and could be calculated for lakes that we had not visited. Extrapolating our results nationwide demonstrated that nearly all of the least disturbed lakes are already of conservation interest because they host small populations of endangered birds [28]. Using well known sites to calibrate the scale suggest that extreme degradation may be more common than has been noted in the literature. Alaotra is one of the only major wetlands in Madagascar that has been subject to conservation and research interest, but this has not stopped the degradation at the site worsening [32]. Large areas of cultivation are inundated by siltation, the marshes that might have sequestered the sediment are burned, organic pollution is leading to eutrophication, and overfishing is decreasing fish yields [32,33]. Our results show that this level of degradation is not exceptional, as Alaotra was far from the worst wetland on our habitat disturbance axis.

There was a notable exception to this otherwise gloomy picture, a predicted good quality site for which no information was available. As a consequence of this modelling outcome we visited the site, located on the boundary between Betsiboka, Boeny and Melaky Regions, in November 2016. We found large populations of several endemic birds of conservation concern, including the Malagasy race of White-backed duck (*Thalassornis leuconotus insularis*), Madagascar grebe and Madagascar jacana (*Actophilornis albinucha*). This finding is a welcome bonus from this research, and demonstrates the validity and utility of our modelling approach. However, this is a fairly small site and our results suggest that it is the only high-quality wetland in Madagascar that the scientific and conservation communities were unaware of.

The status of wetlands globally is generally very poorly known [1,4,34], so it is difficult to put our results in a global context. Some figures do stand out: for example, our estimate that 82% of marsh habitat has been converted to agriculture at our study sites is comparable to estimates of wetland destruction in densely populated regions of Europe and Asia [34] and therefore astonishing for such a sparsely populated country as Madagascar. We can conclude that the only near-pristine lakes in Madagascar are very small and remote sites. Lakes that have remained in good condition are largely the consequence of remoteness from human activity and not of any conservation management. Overall it seems reasonable to say that, despite very incomplete knowledge of threats, far more conservation effort should be directed at wetlands globally [6] and our results confirm this in Madagascar. However, it is not clear what form that effort should take. Effective conservation of freshwater systems is a very recent scientific debate, complicated by the fact that freshwater systems may drain land far beyond the freshwater feature of conservation interest [35]. This means there are, as yet, no clear answers for practitioners, leaving conservationists to make pragmatic judgements based on local laws and processes. Furthermore, conserving Madagascar’s wetlands in their present condition might arrest the declines observed in threatened endemic species but such an approach offers no scope for populations to recover.

### The future of wetland conservation in Madagascar

With almost no wetland protected areas and a wetland dependent population, Community-Based Conservation (CBC) is the only option open to practitioners who wish to conserve wetland biodiversity in Madagascar. There are a small number of ongoing wetland conservation projects. However, several recent studies have questioned the success of CBC in Madagascar, citing a lack of realistic sustainable funding mechanisms for the failures [36–41]. Communities expect conservation to deliver economic benefits [39] but it rarely can. Ecotourism has been relied on to fund projects, but limited tourist numbers, poor infrastructure and political problems mean that few sites make a profit [42,43]. Long-term external funding has been known to work [39,44], but is difficult to secure and subsequently unviable for most sites. In an attempt to find sustainable funding sources, economic incentives related to natural resource extraction have been tried but there have been few success stories. In general, community resource management groups fail, either because of restrictions put on resource use constraining the ability to generate benefits [39] or because the resources on which management is based are marginal economically [40,41].

Notable exceptions to this failure of community-based natural resource management come from marine and freshwater systems [40,41]. This may be because the resources in such systems are less marginal economically, and management of such resources provides genuine improvements to livelihoods. Until recently there have been few projects focused on freshwater or marine systems, and the question has not been systematically reviewed, so it is hard to say how generally this might apply.

It has been suggested that where wetland community-based natural-resource management projects have succeeded it has been because the conservation agency involved and the community are interested in the same goal [41], usually of increasing fish stocks, and that that goal is of economic importance. This might suggest that wetland conservation where this is not true will not work, if, for example, other wetland resources suffer the same problem as forest resources of being economically marginal. This is an important question to answer given the obvious degradation suffered by wetlands worldwide, but as the economic usefulness of any resource may vary between cultures, results from one country or even region may not be applicable elsewhere.

Several freshwater conservation projects have been started relatively recently under legislation introduced in 2003 that aimed to triple the protected areas in Madagascar [e.g. 28]. This legislation does not remove the need for CBC, as these new protected areas are not strictly protected and therefore allow people to live in them and utilize natural resources [40]. These projects are generally aiming to preserve high biodiversity-value remnants. We suggest that this approach may deliver only relatively small benefits for biodiversity and livelihoods, as even these remnants are degraded sites and often host only small populations of endangered wildlife. Large-scale restoration of degraded wetlands may therefore be an appropriate target for conservation agencies. With our current state of knowledge, this will involve trying to manage all forms of human disturbance at wetlands–requiring an expensive, watershed level approach, focusing on increasing agricultural productivity in an ecologically efficient way to meet the demands of a growing population [7]–but the prize of boosting both wildlife and ecosystem services to local people is perhaps worth the effort.

Given how little research and conservation attention tropical wetlands have received, progress from here will involve answering several questions. Most important is to disentangle the effects of various human pressures on biodiversity and whether effects are similar between taxa. Invertebrate surveys with species-level identification may go some way to answering this, as individual species responses to disturbance may reveal more about which pressures are limiting the invertebrate community. However, there are very few sites in good condition to use in comparisons, so fully answering this question will involve either experimental manipulations or a paleo-ecological approach, using sediment cores to reconstruct the history of wetlands [45]. Once this is known, we need to identify what management interventions will most benefit wildlife and second, what community management projects will most benefit rural livelihoods. None of this research will achieve much by itself, however, while the institutional capacity to effect change remains so low in tropical countries [8].

## Supporting information

S1 Table Human disturbance and biodiversity data for the 37 lakes visited during our surveys. (XLSX)

S2 Table Results from PCA analyses of human disturbance data. Tables showing loadings and importance for the nine variables in the full disturbance dataset, and for the five variables in the GIS derived subset.
(DOCX)

S3 Table Country-wide wetland status. Locations and disturbance variables for 973 wetlands predicted by GIS and confirmed against Landsat images.
(XLSX)

S4 Table Endemic bird surveys at independent samples of 22 lakes. (XLSX)
