# Supplementary material for: Profound and pervasive degradation of Madagascar’s freshwater wetlands and links with biodiversity
Source: PLoS One. 2017 Aug 8;12(8):e0182673. doi: 10.1371/journal.pone.0182673 (PMC5549726; doi:10.1371/journal.pone.0182673)
Supplement: S2 Table — Tables showing loadings and importance for the nine variables in the full disturbance dataset, and for the five variables in the GIS derived subset. (DOCX) [file pone.0182673.s002.docx]

S2 Table (A) Loadings and importance of Principal Components from analysis of human disturbance variables at wetland sites in Madagascar.

|  | Component | | | |
| --- | --- | --- | --- | --- |
|  | 1 | 2 | 3 | 4 |
| *Loadings* |  |  |  |  |
| Sedimentation | -0.34 | -0.14 | 0.32 | -0.65 |
| Marsh clearance | -0.35 | -0.27 | -0.42 | 0.02 |
| Rice / lake | -0.27 | -0.30 | -0.62 | 0.10 |
| Rice in watershed | -0.22 | -0.50 | 0.28 | 0.01 |
| Invasive species | -0.42 | -0.06 | 0.03 | -0.25 |
| Forest clearance in watershed | -0.37 | 0.04 | 0.43 | 0.37 |
| Forest clearance around lake | -0.39 | 0.09 | 0.16 | 0.58 |
| Population density in watershed | -0.27 | 0.55 | -0.07 | -0.16 |
| Population density near lake | -0.32 | 0.50 | -0.61 | -0.09 |
|  |  |  |  |  |
| *Importance* |  |  |  |  |
| St. Dev | 2.12 | 1.33 | 1.04 | 0.79 |
| Proportion of variance | 0.50 | 0.20 | 0.12 | 0.07 |
| Cumulative proportion | 0.50 | 0.70 | 0.82 | 0.89 |

S2 Table (B) Loadings and importance of principal components from analysis of a reduced set of human disturbance variables at wetland sites in Madagascar.

|  | Component | | | |
| --- | --- | --- | --- | --- |
|  | 1 | 2 | 3 | 4 |
| *Loadings* |  |  |  |  |
| Sedimentation | -0.35 | 0.49 | 0.76 | 0.22 |
| Forest clearance in watershed | -0.47 | 0.42 | -0.26 | -0.64 |
| Forest clearance around lake | -0.48 | 0.22 | -0.55 | 0.52 |
| Population density in watershed | -0.45 | -0.52 | 0.18 | -0.39 |
| Population density near lake | -0.47 | -0.51 | 0.07 | 0.32 |
|  |  |  |  |  |
| *Importance* |  |  |  |  |
| St. Dev | 1.75 | 1.04 | 0.77 | 0.43 |
| Proportion of variance | 0.62 | 0.22 | 0.12 | 0.04 |
| Cumulative proportion | 0.62 | 0.83 | 0.95 | 0.99 |

|  |  | *Anas melleri* | | *Tachybaptus pelzelnii* | |
| --- | --- | --- | --- | --- | --- |
| Model | d.f. | AIC | Akaike weight | AIC | Akaike weight |
| Constant occupancy and detection | 2 | 42.8 | 0.10 | 30.4 | 0.00 |
| Occupancy alters with PC1, constant detection | 3 | **40.2** | **0.37** | 23.0 | 0.18 |
| Constant occupancy, detection alters with PC1 | 3 | 42.0 | 0.15 | 22.2 | 0.28 |
| Occupancy and detection alter with PC1 | 4 | **40.1** | **0.39** | **20.9** | **0.53** |

Table S3. AIC values for patch occupancy models of two bird species at 15 lakes that were repeatedly surveyed. The best model is highlighted in bold for each species.
